# Systemic inflammatory biomarkers (NLR, SII, PNI and FPR) combined with CEA for predicting advanced colorectal neoplasms: development and temporal validation of a machine learning model

**DOI:** 10.3389/fonc.2026.1832546

**Published:** 2026-07-22

**Authors:** Chang Zhang, Shihao Wu, Liang Lu, Shui Jin

**Affiliations:** 1 Department of Gastroenterology, The Fourth Affiliated Hospital of Anhui Medical University, Hefei, China; 2 Department of Burns, The First Affiliated Hospital of Anhui Medical University, Hefei, China

**Keywords:** advanced colorectal neoplasms, machine learning, neutrophil-to-lymphocyte ratio, systemic immune-inflammation index, systemic inflammatory biomarkers

## Abstract

**Background:**

Chronic systemic inflammation is closely associated with the initiation and development of colorectal tumors. Biomarkers reflecting inflammatory status may help detect advanced colorectal neoplasms (ACRN), which include advanced adenoma and colorectal cancer. Nevertheless, the diagnostic ability of combined inflammatory and nutritional indicators for ACRN has not been fully clarified. This study evaluated whether several systemic inflammation markers—NLR, SII, PNI, and FPR—together with CEA, can improve the identification of ACRN.

**Methods:**

A retrospective analysis was conducted in individuals who received colonoscopy from December 2016 to December 2024. Eligible subjects were randomly assigned to a training set and an internal testing set. Patients enrolled between January 2025 and January 2026 were used as a temporal validation cohort. Correlation analysis together with restricted cubic spline (RCS) modeling was applied to assess the associations between inflammatory markers and ACRN risk. Machine learning approaches were then used to construct prediction models based on NLR, SII, PNI, FPR, and CEA. Model performance was assessed by discrimination ability, calibration, and clinical usefulness. An online calculator was also established to support individualized risk estimation.

**Results:**

A total of 1330 individuals were analyzed, including 252(18.95%) cases diagnosed with ACRN. Higher values of NLR, SII, FPR, and CEA were linked to a greater probability of ACRN, while PNI showed a negative relationship. These indicators also displayed clear changes across different stages of disease. Among the evaluated machine learning approaches, the XGBoost model showed the strongest predictive ability. The AUCs reached 0.960 in the training set, 0.944 in the testing set, and 0.908 in the temporal validation cohort. In addition, a web-based calculator was built to support personalized risk assessment.

**Conclusion:**

Systemic inflammatory and nutritional biomarkers combined with CEA demonstrated robust predictive performance for identifying advanced colorectal neoplasms and may provide a convenient tool for early risk stratification in colorectal cancer screening.

## 1 Introduction

Colorectal cancer (CRC) is the third most frequently diagnosed malignancy and ranks second among causes of cancer-related death worldwide (1–3). In China, both incidence and mortality have shown a steady rise in recent years, creating a growing public health burden (4, 5). CRC usually develops through the classic adenoma–carcinoma pathway (6, 7), evolving from normal mucosa to adenoma and finally carcinoma over many years. This long disease course offers an important opportunity for early detection and timely intervention. Advanced colorectal neoplasms (ACRN), including advanced adenomas (AA) and colorectal cancer, are key targets in CRC screening because they are strongly linked to later cancer development (8, 9). Epidemiological evidence indicates that individuals with advanced adenomas face a 20–30% chance of progressing to CRC within five years (8, 10). Detecting and removing these lesions at an early stage can markedly lower CRC incidence and related mortality (11). Current screening methods still show several limitations. Fecal occult blood testing has limited sensitivity (12–14), whereas colonoscopy, although regarded as the gold standard (15), is invasive, requires bowel preparation, and patient adherence is often suboptimal (16, 17). For this reason, simple, noninvasive, and dependable biomarkers for early identification and risk stratification of ACRN are of clear clinical value.

Growing evidence suggests that persistent systemic inflammation contributes to the development and progression of colorectal tumors (18–20). Inflammatory activity can support tumor formation through several pathways, such as immune imbalance, oxidative stress, cytokine production, and changes in the tumor microenvironment (21, 22). In recent years, biomarkers derived from routine blood tests have attracted attention as indicators of systemic inflammatory status. Common examples include the neutrophil-to-lymphocyte ratio (NLR), systemic immune-inflammation index (SII), prognostic nutritional index (PNI), and fibrinogen-to-prealbumin ratio (FPR). These indices reflect the interaction among inflammatory activity, immune response, and nutritional condition. Previous studies have reported that such markers may provide useful diagnostic or prognostic information in several cancers, including CRC.

Earlier research has indicated that single inflammatory indicators may help in detecting CRC.However, several shortcomings still exist. Most studies have focused primarily on CRC itself while overlooking advanced adenomas, a critical precancerous stage (23–25). Moreover, traditional statistical approaches may have limited ability to capture complex nonlinear relationships among multiple biomarkers. Machine learning algorithms provide powerful tools for integrating multidimensional data and identifying hidden patterns within complex datasets, thereby offering improved predictive performance in clinical risk assessment (26, 27).

This study assessed the predictive ability of several systemic inflammatory and nutritional indicators, including NLR, SII, PNI, and FPR, together with carcinoembryonic antigen (CEA), for detecting advanced colorectal neoplasms. Machine learning methods were further used to build and validate prediction models. In addition, a web-based calculator was created to allow personalized risk estimation and to assist early detection in clinical settings.

## 2 Methods

### 2.1 Study participants

Patients who underwent colonoscopy at the Fourth Affiliated Hospital of Anhui Medical University between December 2016 and January 2026 were part of this study. Participants had to meet the following criteria (1): be at least 18 years old (2); have a pathological diagnosis of normal colorectal mucosa, colorectal adenoma, or colorectal adenocarcinoma; and (3) possess complete clinical documentation. Exclusion was based on (1): concomitant malignancies other than colorectal cancer (2); prior history of colorectal surgery (3); presence of acute infection (4); comorbid chronic inflammatory diseases or autoimmune disorders (5); hematological diseases; and (6) recent use of immunomodulatory agents, corticosteroids, or other immunosuppressive therapies.

Colonoscopy in the included population was performed for multiple clinical indications, including gastrointestinal symptoms (such as abdominal pain, change in bowel habits, rectal bleeding, unexplained weight loss, or chronic diarrhea), positive fecal occult blood test or other stool-based screening tests, laboratory abnormalities such as anemia, or participation in opportunistic colorectal cancer screening programs.

According to colonoscopic and pathological findings, patients were classified into three groups: the normal control group (no abnormalities or only non-adenomatous polyps detected by colonoscopy), the AA group (adenomas ≥10 mm in diameter, villous component ≥25%, or high-grade dysplasia) (28, 29), and the CRC group (pathologically confirmed adenocarcinoma without distant metastasis). For the primary outcome, patients with AA and CRC were combined and defined as having ACRN (30). The normal control group consisted of individuals who underwent colonoscopy for heterogeneous clinical indications, including both screening-related examinations (population-based or opportunistic colorectal cancer screening) and evaluation of non-specific gastrointestinal symptoms. Importantly, all individuals in this group had completely normal colonoscopic findings or only non-neoplastic lesions, such as hyperplastic polyps, and no clinically relevant colorectal neoplasia was identified.

### 2.2 Data collection and biomarker calculation

Data on demographic traits and lab test outcomes were sourced from the electronic medical records. All lab tests were conducted using fasting venous blood samples taken within a week prior to the colonoscopy.

Demographic variables included age. Tumor markers included CEA, carbohydrate antigen 19-9 (CA199), alpha-fetoprotein (AFP), carbohydrate antigen 125 (CA125), carbohydrate antigen 153 (CA153), and carbohydrate antigen 724 (CA724). Biochemical parameters included fibrinogen (FIB), calcium (Ca), urea, creatinine, alanine aminotransferase (ALT), aspartate aminotransferase (AST), total protein (TP), albumin (ALB), and albumin-to-globulin ratio (A/G). Hematological parameters included red blood cell count, hemoglobin (Hb), mean corpuscular hemoglobin concentration (MCHC), neutrophil count, lymphocyte count, monocyte count, and platelet count (PLT).

Based on these variables, several derived inflammatory and nutritional indicators were calculated:

Neutrophil-to-lymphocyte ratio (NLR) = neutrophil count/lymphocyte count

Platelet-to-lymphocyte ratio (PLR) = platelet count/lymphocyte count

Systemic immune–inflammation index (SII) = platelet count × neutrophil count/lymphocyte count

Prognostic nutritional index (PNI) = albumin (g/L) + 5 × lymphocyte count (×10^9^/L)

Fibrinogen-to-prealbumin ratio (FPR) = fibrinogen (g/L)/prealbumin (g/L)

Hemoglobin-to-red blood cell ratio (HRR) = hemoglobin (g/L)/red blood cell count (×10¹²/L)

Hemoglobin-to-platelet ratio (HPR) = hemoglobin (g/L)/platelet count (×10^9^/L)

Lymphocyte-to-monocyte ratio (LMR) = lymphocyte count/monocyte count

Monocyte-to-lymphocyte ratio (MLR) = monocyte count/lymphocyte count

### 2.3 Statistical analysis

R software (version 4.3.2) was used for all statistical analyses. Missing data (<5%) were handled using multiple imputation by chained equations (MICE) with the mice package, generating five datasets over 10 iterations. Continuous variables were imputed using predictive mean matching, and categorical variables were imputed using logistic regression. Missing data proportions are shown in **Supplementary Table 1**, and results were pooled according to Rubin’s rules. The Shapiro-Wilk test was used to assess the normality of continuous variables. For comparisons between two groups, normally distributed continuous variables were expressed as the mean ± standard deviation and compared using independent-samples t-tests. Non-normally distributed variables were expressed as the median (interquartile range) and compared using the Mann-Whitney U test. Categorical variables were presented as numbers (percentages) and compared using the chi-square test or Fisher’s exact test, as appropriate. To control for multiple comparisons in baseline analyses, P values were adjusted using the Holm method. Initially, univariate logistic regression was conducted in the training set to identify variables associated with ACRN (P < 0.05). To prevent overfitting and select reliable predictors, we used two complementary machine learning-based feature selection methods. The final predictors were defined as the intersection of variables selected by LASSO regression with 10-fold cross-validation and the Boruta algorithm, which is based on random forests. Spearman correlation analysis was used to assess relationships among the selected biomarkers. The variance inflation factor (VIF) was calculated to evaluate multicollinearity, with VIF > 5 indicating substantial collinearity (31). Restricted cubic spline (RCS) analysis was used to investigate potential nonlinear relationships between major inflammatory biomarkers and ACRN risk (32). To evaluate potential confounding by metabolic comorbidities, we compared the distribution of diabetes mellitus, cardiovascular disease, and body mass index categories between the non-advanced and ACRN groups using the chi-square test. Multivariable logistic regression analysis was then performed to assess the independent associations of the five selected biomarkers with ACRN after adjustment for these metabolic factors, with odds ratios and 95% confidence intervals calculated for each variable. To evaluate monotonic biomarker trends across the normal, adenoma, and CRC groups, the Jonckheere-Terpstra trend test was applied and visualized with box plots. All statistical tests were two-sided, and P < 0.05 was considered statistically significant.

### 2.4 Machine learning model development and validation

Seven machine learning algorithms, including Random Forest (RF), Classification and Regression Tree (CART), Gradient Boosting Machine (GBM), k-nearest neighbors (KNN), Neural Network (NNet), and Extreme Gradient Boosting (XGBoost), were used to develop prediction models based on the selected predictors. To minimize model selection bias, models were trained using 10-fold cross-validation repeated 10 times. Model performance was assessed using the area under the receiver operating characteristic curve (AUC), accuracy, sensitivity, specificity, precision, Kappa, Matthews correlation coefficient (MCC), F1 score, and Brier score. Agreement between predicted and observed outcomes was assessed using Cohen’s Kappa coefficient. Kappa values range from -1 to 1, with values >0.75 indicating strong agreement, 0.40-0.75 indicating moderate agreement, and <0.40 indicating poor agreement (33). MCC was used as a balanced performance metric that accounts for all four elements of the confusion matrix and is suitable for imbalanced datasets. MCC ranges from -1 to 1, with higher values indicating better predictive performance (34). The F1 score, the harmonic mean of precision and recall, was used to measure model robustness (35). Calibration was evaluated using the Brier score, with lower values indicating better calibration. Decision curve analysis (DCA) was conducted to evaluate the clinical utility of the prediction models. To further evaluate whether the model’s high discriminative performance was driven primarily by colorectal cancer cases, we performed stratified receiver operating characteristic (ROC) analyses separately for advanced adenoma and colorectal cancer subgroups, using the non-advanced group as the common reference. The area under the ROC curve (AUC) with 95% confidence intervals was calculated using the bootstrap method with 1, 000 resamples for each subgroup analysis. The best-performing final model was analyzed using SHapley Additive exPlanations (SHAP) to quantify the contribution of each predictor to model output (36). R packages including caret, randomForest, xgboost, e1071, nnet, Boruta, rms, pROC, mice, and SHAPforxgboost were used for statistical analyses and model development.

## 3 Results

### 3.1 Baseline characteristics of the study participants

**Figure 1:**
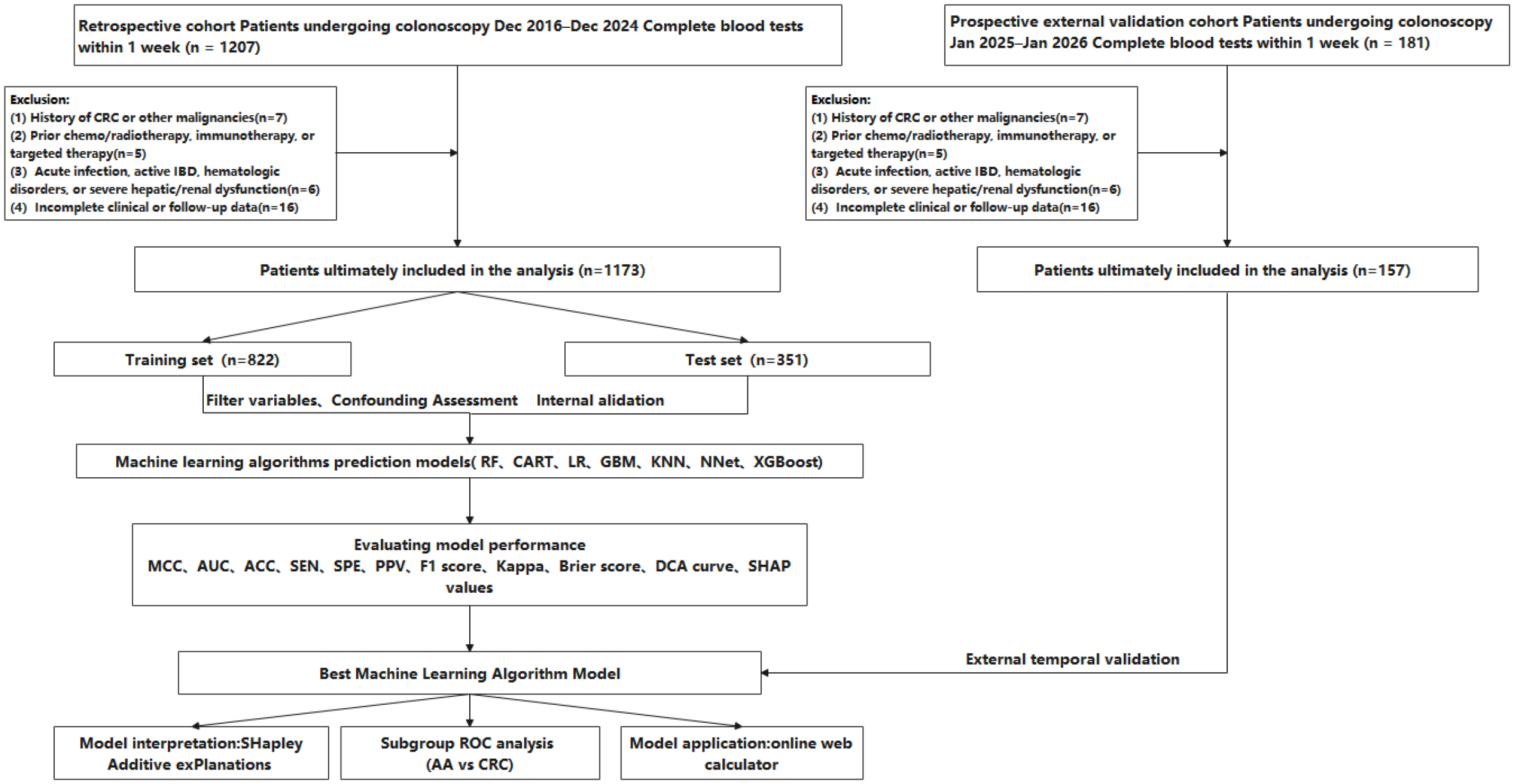
Study flowchart.

After applying the inclusion and exclusion criteria, a total of 1330 participants were included in the final analysis, including 252 patients with advanced colorectal neoplasia (ACRN) and 1078 non-ACRN cases (normal mucosa or non-advanced adenomas). The dataset was randomly divided into a training cohort (n = 822, including 147 ACRN cases) and an internal testing cohort (n = 351, including 63 ACRN cases) at a 7:3 ratio. In addition, an independent temporal validation cohort (n = 157, including 42 ACRN cases) collected between January 2025 and January 2026 was used for external validation. No significant differences in baseline characteristics were observed between the training and testing cohorts. A total of 28 candidate biomarkers were extracted from each participant for subsequent model development. **Table 1** summarizes the baseline clinical characteristics of the training cohort, while **Supplementary Table 2** presents those of the temporal validation cohort. The distribution of ACRN cases across the training, testing, and validation cohorts was clearly defined, ensuring transparency and robustness of model evaluation. The study selection and analysis workflow are illustrated in **Figure 1**.

**Table 1:** Baseline characteristics of the training cohort.

Variables	Non-advanced group (n=675)	Advanced group (147)	P-value
Age[Median (Q1-Q3), years]	58.00 [51.00, 66.50]	67.00 [56.00, 71.00]	<0.001
Ca[Median (Q1-Q3), mmol/L]	2.32 [2.00, 2.41]	2.31 [2.02, 2.45]	1.000
Urea[Median (Q1-Q3), mmol/L]	5.80 [4.80, 6.80]	5.90 [4.90, 7.40]	0.961
Creatinine[Median (Q1-Q3), μmol/L]	69.00 [58.00, 84.00]	68.00 [56.60, 82.40]	1.000
FIB[Median (Q1-Q3), g/L]	2.75 [2.38, 3.14]	2.87 [2.20, 3.63]	1.000
AFP[Median (Q1-Q3), ng/mL]	2.50 [1.86, 3.39]	2.61 [1.71, 3.72]	1.000
CEA[Median (Q1-Q3), ng/mL]	2.20 [1.52, 3.15]	11.00 [2.41, 13.64]	<0.001
CA199[Median (Q1-Q3), U/mL]	3.74 [2.00, 6.64]	16.00 [2.05, 36.34]	<0.001
CA125[Median (Q1-Q3), U/mL]	10.70 [8.20, 14.70]	15.00 [10.80, 22.00]	<0.001
CA153[Median (Q1-Q3), U/mL]	8.30 [6.20, 11.90]	9.60 [6.80, 13.65]	0.495
CA724[Median (Q1-Q3), U/mL]	1.72 [1.02, 2.93]	1.89 [0.55, 3.83]	1.000
ALT[Median (Q1-Q3), U/L]	22.00 [16.00, 31.00]	23.00 [15.00, 35.50]	1.000
AST[Median (Q1-Q3), U/L]	23.00 [19.00, 27.00]	25.00 [17.00, 33.00]	0.961
TP[Median (Q1-Q3), g/L]	72.00 [67.75, 75.50]	70.20 [61.90, 73.05]	<0.001
ALB[Median (Q1-Q3), g/L]	45.90 [43.10, 48.00]	37.00 [28.80, 42.75]	<0.001
A/G	1.77 [1.58, 1.92]	1.62 [1.29, 1.95]	0.002
RBC[Median (Q1-Q3), ×10¹²/L]	4.67 [4.35, 5.00]	4.14 [3.64, 4.98]	<0.001
Hb[Median (Q1-Q3), g/L]	135.00 [115.00, 145.00]	127.00 [111.50, 144.00]	0.961
MCHC[Median (Q1-Q3), g/L]	332.00 [326.00, 338.00]	336.00 [328.00, 345.50]	0.007
NLR[Median (Q1-Q3)]	2.03 [1.57, 2.97]	5.32 [3.00, 5.95]	<0.001
PLR[Median (Q1-Q3)]	113.54 [83.80, 150.00]	156.02 [92.72, 222.00]	<0.001
PNI[Median (Q1-Q3)]	55.10 [51.83, 59.12]	35.40 [31.48, 52.23]	<0.001
SII[Median (Q1-Q3)]	339.90 [193.56, 499.76]	625.17 [379.72, 803.95]	<0.001
FPR[Median (Q1-Q3)]	15.00 [12.00, 19.00]	23.00 [21.00, 26.50]	<0.001
HRR[Median (Q1-Q3)]	8.91 [7.83, 9.92]	11.47 [9.44, 13.00]	<0.001
HPR[Median (Q1-Q3)]	0.74 [0.61, 0.91]	0.76 [0.55, 1.00]	1.000
LMR[Median (Q1-Q3)]	4.34 [2.88, 5.85]	3.34 [2.10, 4.97]	<0.001
MLR[Median (Q1-Q3)]	0.20 [0.17, 0.26]	0.22 [0.17, 0.30]	1.000

^*^ P values were adjusted for multiple comparisons using the Holm method.

### 3.2 Identification of immuno-inflammatory predictors for ACRN

**Figure 2:**
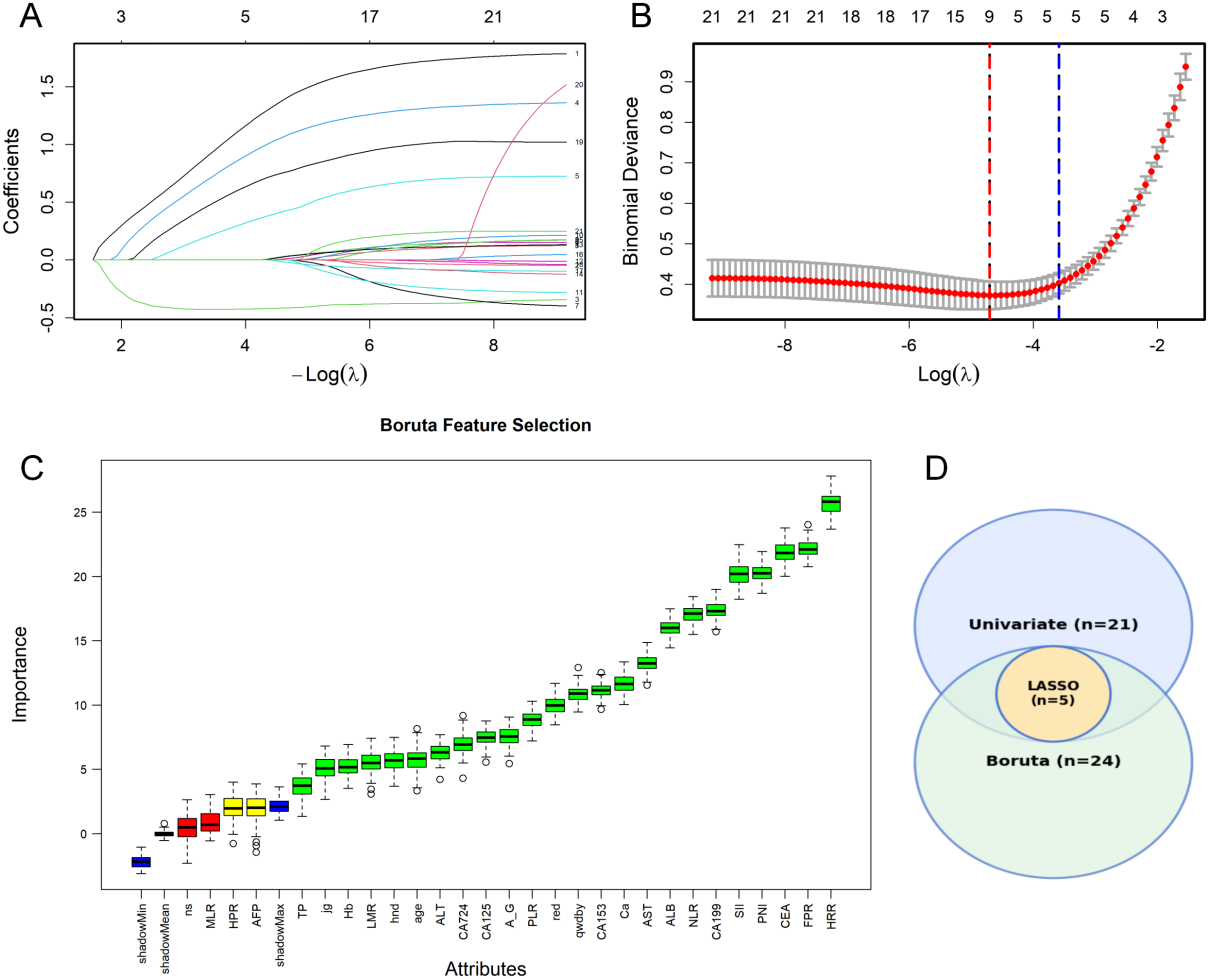
Feature selection of candidate predictors for ACRN. **(A)** Univariate logistic regression.Univariate logistic regression analysis identified 21 variables significantly associated with ACRN. **(B)** LASSO regression.The optimal lambda value (λ) was determined by 10-fold cross-validation, resulting in the selection of 5 non-zero coefficients (CEA, SII, FPR, PNI, NLR). **(C)** Boruta algorithm. Variable importance plot. Green boxplots represent confirmed variables (n = 24), red boxplots represent rejected variables, and yellow boxplots represent tentative variables. The algorithm confirmed 24 variables as important for ACRN diagnosis. **(D)** Venn diagram illustrating the overlap of variables selected by the three methods. The intersection contains 5 common variables (CEA, SII, NLR, PNI, FPR), which were used for subsequent modeling.

A multi-step feature selection process was performed in the training set to identify the strongest predictors of ACRN among 28 candidate peripheral blood markers. The association between each candidate marker and ACRN was first evaluated using univariate logistic regression. Variables with P < 0.05 were then entered into a LASSO regression model for dimensionality reduction. The optimal penalty parameter lambda was determined using 10-fold cross-validation and the minimum deviance criterion. In parallel, the Boruta algorithm, a random forest-based feature selection method, was applied to all candidate variables to identify relevant predictors by comparing the importance of original variables with shadow features (37). The final predictors were defined as variables selected by both LASSO regression and Boruta. This process yielded five key variables: NLR, CEA, SII, PNI, and FPR. These biomarkers reflect systemic immuno-inflammatory status, nutritional immunity, and tumor burden, and were retained for subsequent multivariable modeling (**Figure 2**).

### 3.3 Correlation and multicollinearity analysis of selected biomarkers

**Figure 3:**
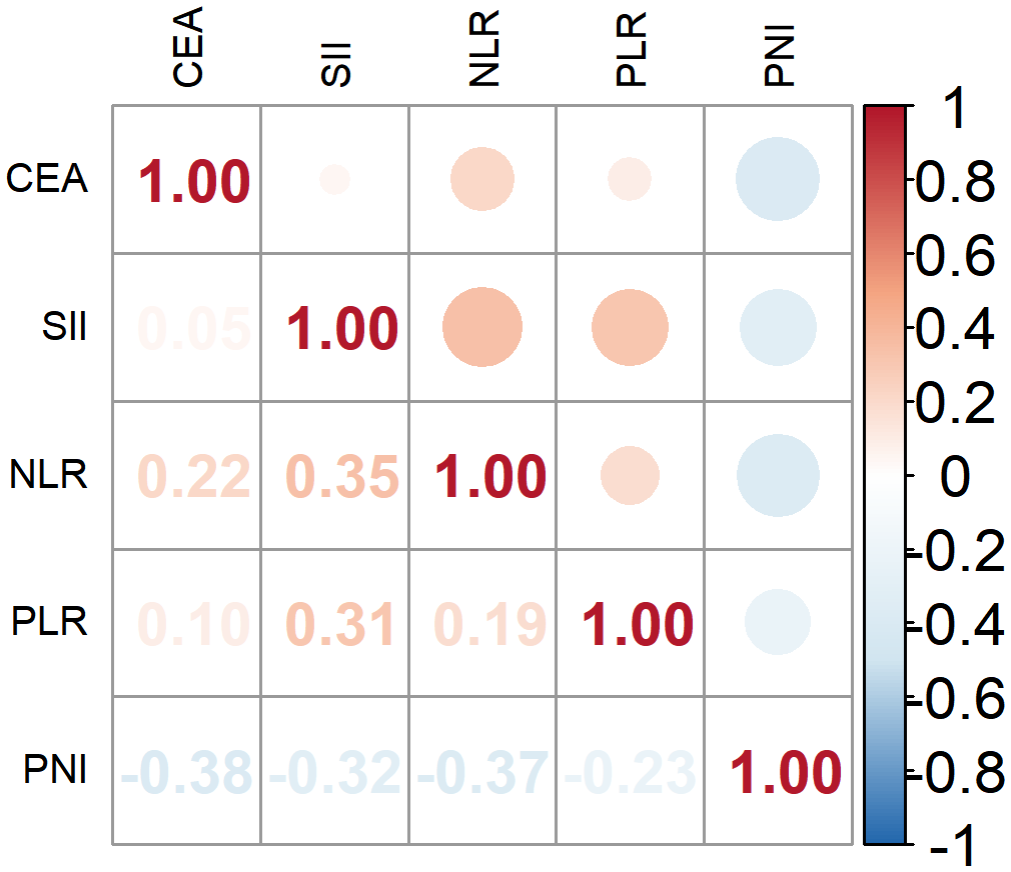
Correlation heatmap of the selected biomarkers. The heatmap illustrates the pairwise correlations among CEA, SII, NLR, PNI, and FPR.

To better characterize the relationships among the selected predictors, Spearman correlation analysis was conducted for CEA, SII, NLR, PNI, and FPR, and the results are shown in a correlation heatmap (**Figure 3**). The analysis showed weak to moderate correlations among these variables, with no strong correlations observed (|r| < 0.4). The VIF was then calculated for each predictor to assess multicollinearity. The VIF values for CEA, SII, NLR, PNI, and FPR were 1.26, 1.22, 1.31, 1.48, and 1.15, respectively, all below the commonly accepted threshold of 5. These results indicate no substantial multicollinearity among the selected predictors and support their combined use in subsequent multivariable models.

### 3.4 Nonlinear dose-response relationships

**Figure 4:**
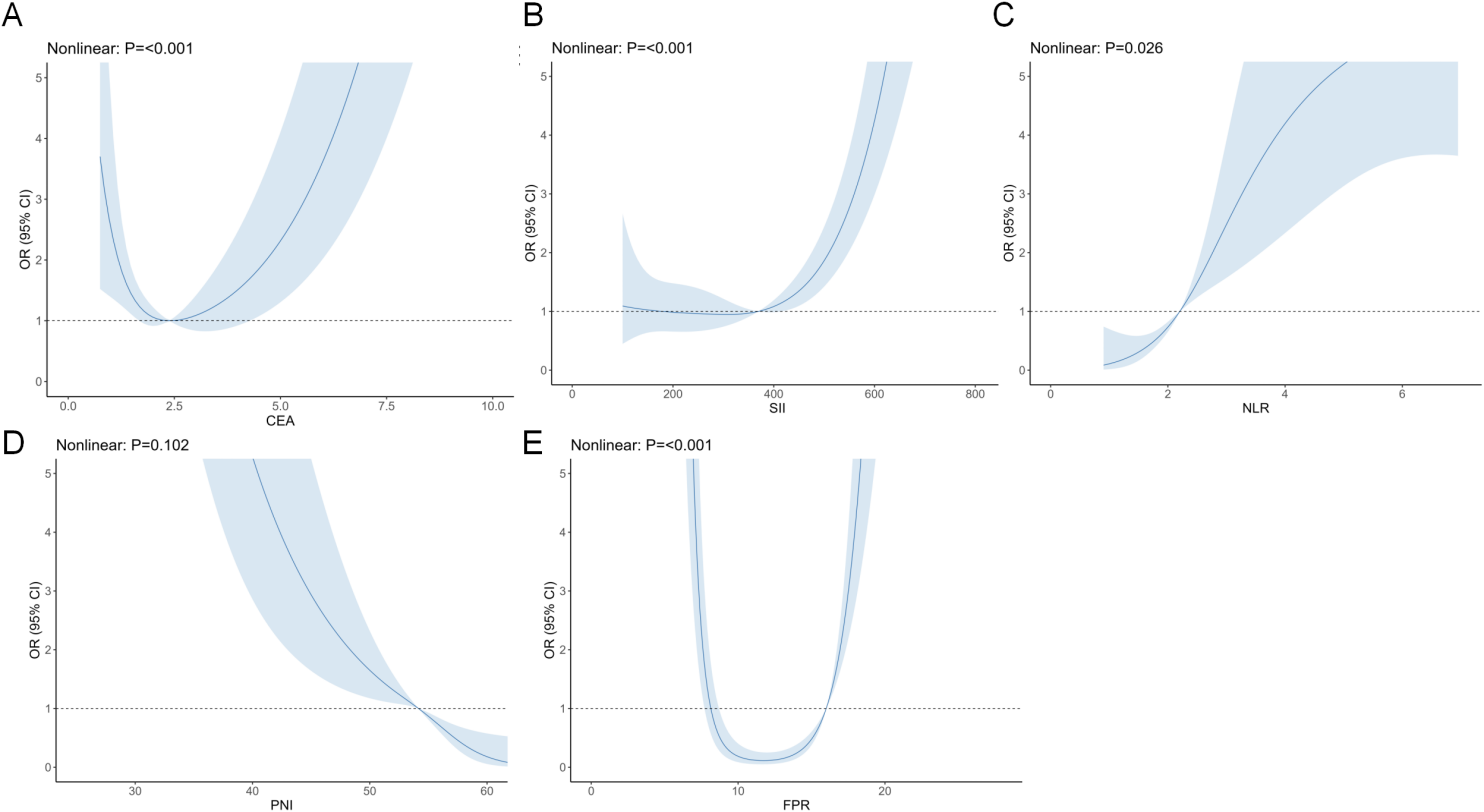
Restricted cubic spline analysis of inflammatory biomarkers and ACRN risk. **(A)** CEA. **(B)** SII. **(C)** NLR. **(D)** PNI. **(E)** FPR. RCS models were used to evaluate the dose–response relationships between inflammatory biomarkers and the risk of ACRN. The solid blue lines represent the estimated odds ratios (ORs), and the shaded areas indicate the 95% CI. The dashed horizontal line indicates OR = 1 as the reference level.

To investigate dose-response relationships between major inflammatory and nutritional biomarkers and ACRN risk, RCS models were used to assess potential nonlinear associations for CEA, SII, NLR, FPR, and PNI (**Figure 4**). RCS analysis showed significant nonlinear associations between CEA, SII, NLR, and FPR and ACRN risk (nonlinear P < 0.001). In contrast, no significant nonlinear relationship was observed for PNI.

### 3.5 Assessment of confounding by metabolic comorbidities

To evaluate whether common metabolic comorbidities confounded the association between inflammatory biomarkers and ACRN, we compared the distribution of diabetes mellitus (DM), cardiovascular disease (CVD), and body mass index (BMI) between the non-advanced and ACRN groups in the train cohort. As shown in **Supplementary Table 3**, no significant differences were observed in the prevalence of DM, CVD, or BMI categories between the two groups (all P > 0.05). In a multivariable logistic regression model adjusting for these metabolic factors together with the five selected biomarkers, all five biomarkers remained independently associated with ACRN (all P ≤ 0.005), whereas DM, CVD, and BMI showed no significant association (all P > 0.05, **Table 2**). These findings suggest that the observed associations between inflammatory biomarkers and ACRN are robust and not substantially confounded by common metabolic conditions.

**Table 2:** Confounding analysis of metabolic comorbidities on the association between inflammatory biomarkers and ACRN.

Variables	OR	95% CI	P value
NLR	1.391	1.157-1.672	<0.001
SII	1.808	1.550-2.108	<0.001
PNI	0.958	0.930-0.987	0.005
FPR	1.178	1.113-1.246	<0.001
CEA	1.503	1.367-1.653	<0.001
DM (Yes vs No)	1.399	0.639-3.062	0.401
CVD (Yes vs No)	0.600	0.305-1.180	0.138
BMI (underweight vs normal)	1.726	0.890-3.346	0.106
BMI (overweight vs normal)	0.584	0.171-1.996	0.391

### 3.6 Systemic inflammation progression across normal-adenoma-carcinoma sequence

**Figure 5:**
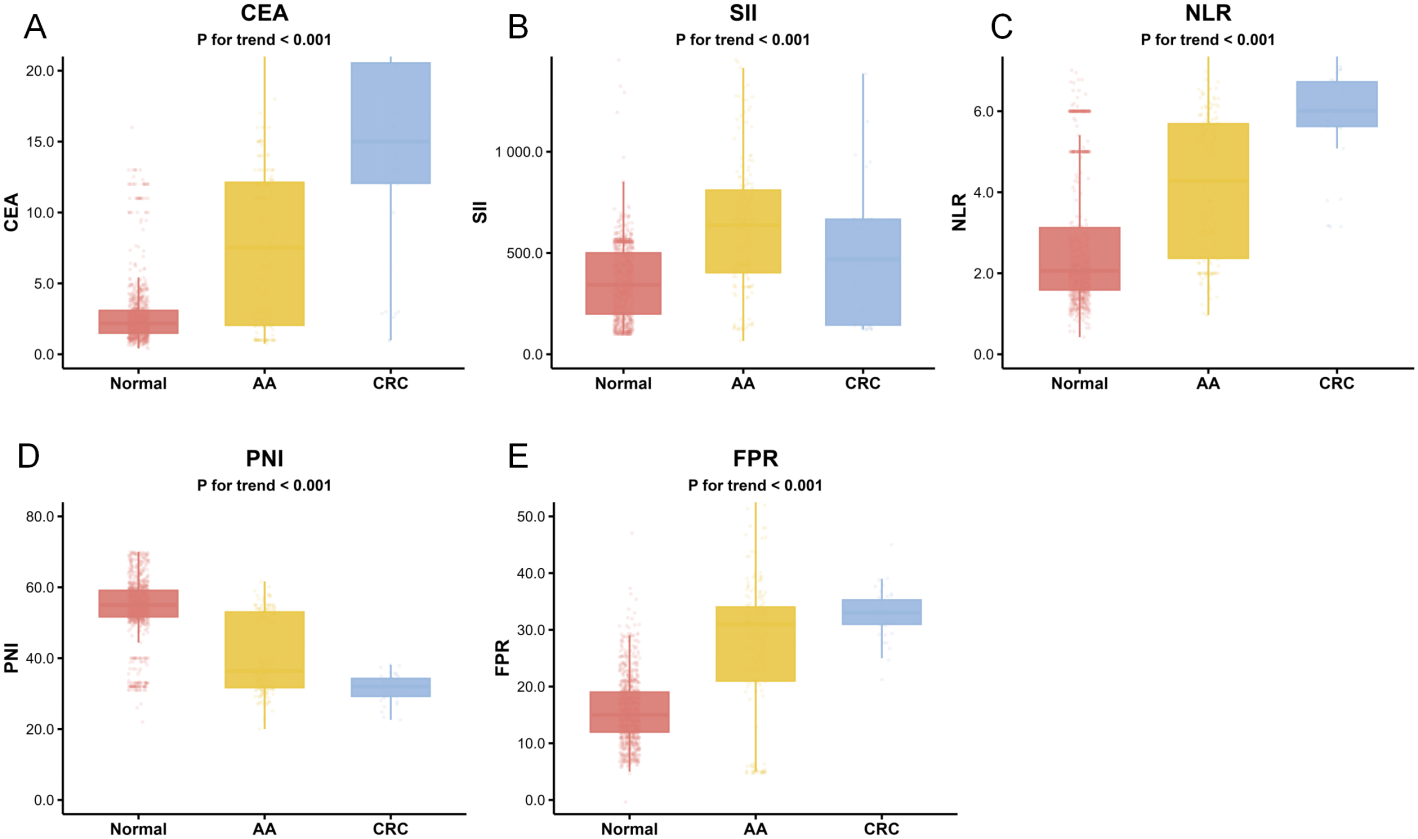
Distribution and trend of inflammatory biomarkers across disease stages. **(A)** CEA. **(B)** SII. **(C)** NLR. **(D)** PNI. **(E)** FPR.Boxplots illustrate the distribution of inflammatory biomarkers among the normal control group, AA group, and CRC group. The Jonckheere–Terpstra test was applied to evaluate ordered trends across the three groups. P values for trend are indicated in each panel.

To evaluate the dynamic changes of inflammatory biomarkers during colorectal tumor progression, the study population was categorized into three groups according to clinical outcomes: normal controls, AA, and CRC. To examine ordered differences across these groups, the Jonckheere–Terpstra (JT) trend test was employed. As shown in **Figure 5**, all five biomarkers exhibited significant monotonic trends across disease stages (P for trend < 0.001) (38). Specifically, NLR, FPR, SII, and CEA exhibited stepwise escalation from normal mucosa to AA and further to CRC, reflecting progressive activation of innate immunity and chronic systemic inflammation. Conversely, PNI demonstrated a significant declining trend, indicating deteriorating nutritional immunocompetence and lymphocyte-mediated anti-tumor immunity along the malignant continuum. Notably, CRC patients exhibited the highest levels of NLR, FPR, SII, and CEA but the lowest PNI, underscoring the critical role of systemic immune dysregulation in driving colorectal tumorigenesis. These findings support the utility of these biomarkers as indicators of immunological progression during the normal-adenoma-carcinoma transition.

### 3.7 Performance and validation of machine learning models

#### 3.7.1 Internal validation: performance in training and test sets

**Figure 6:**
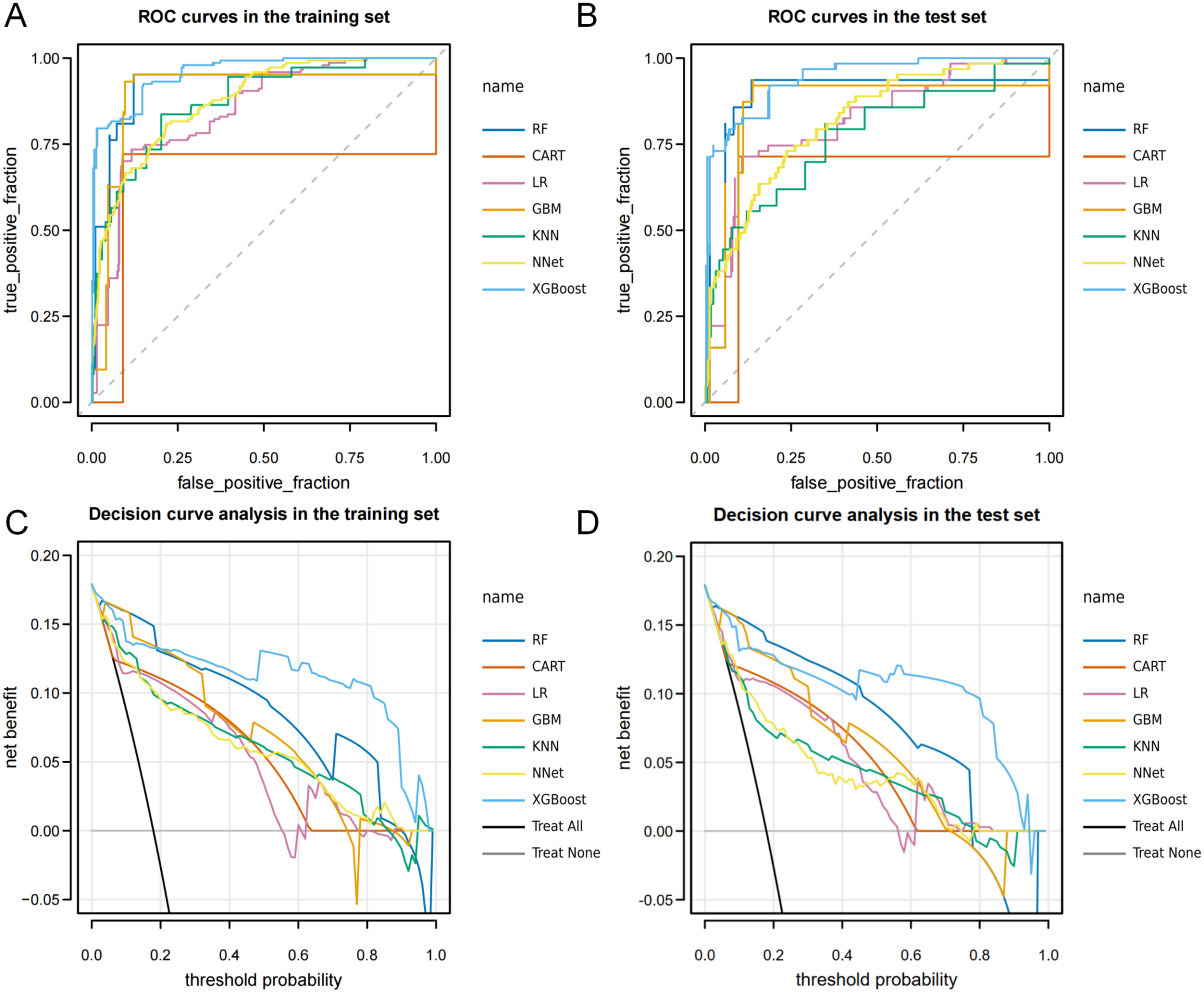
Performance comparison of machine learning models in the training and test cohorts. **(A)** ROC curves of different machine learning models in the training cohort. **(B)** ROC curves in the test cohort. **(C)** DCA of different models in the training cohort. **(D)** DCA in the test cohort.The ROC curves compare the discriminative performance of multiple machine learning algorithms, including RF, CART, LR, GBM, KNN, NNet, and XGBoost. Decision curve analysis evaluates the clinical net benefit of each model across a range of threshold probabilities.

In the training cohort, the XGBoost model achieved the highest predictive performance, with an AUC of 0.960 (95% CI: 0.944–0.975). The model demonstrated an accuracy of 0.951, sensitivity of 0.796, specificity of 0.985, F1 score of 0.854, MCC of 0.828, and a Brier score of 0.084 (**Table 3**). The RF and GBM models also showed strong discriminative ability, with AUC values of 0.946 and 0.927, respectively, although their overall performance metrics were slightly lower than those of the XGBoost model. In the testing cohort, the performance of all models showed a slight decline. However, the XGBoost model maintained the highest predictive performance, with an AUC of 0.944, accuracy of 0.935, sensitivity of 0.746, specificity of 0.976, F1 score of 0.803, MCC of 0.768, and a Brier score of 0.086. By comparison, the AUC values of the RF and GBM models were 0.933 and 0.910, respectively, whereas the CART, LR, KNN, and NNet models achieved AUC values below 0.83 (**Table 3**). The ROC curves for all models in the training and testing cohorts are presented in **Figures 6A, B**, respectively. In addition, DCA demonstrated that the XGBoost model provided greater net clinical benefit across a wide range of threshold probabilities compared with the other models (**Figures 6C, D**). Overall, these results indicate that the XGBoost model demonstrated the best discriminative ability and calibration performance among all evaluated models in both the training and testing cohorts.

**Table 3:** Predictive performance of machine learning models in the training and test cohorts.

Dataset	Model	AUC	ACC	SEN	SPE	Precision	F1	Kappa	Brier	MCC
Train	RF	0.946	0.892	0.952	0.878	0.631	0.759	0.693	0.062	0.717
	CART	0.815	0.876	0.721	0.910	0.635	0.675	0.599	0.094	0.601
	LR	0.853	0.876	0.721	0.910	0.635	0.675	0.599	0.146	0.601
	GBM	0.927	0.909	0.932	0.904	0.678	0.785	0.729	0.146	0.744
	KNN	0.889	0.805	0.836	0.798	0.475	0.606	0.489	0.096	0.524
	NNet	0.877	0.788	0.810	0.783	0.449	0.578	0.451	0.097	0.486
	XGBoost	0.960	0.951	0.796	0.985	0.921	0.854	0.825	0.084	0.828
Test	RF	0.933	0.878	0.937	0.865	0.602	0.733	0.659	0.069	0.686
	CART	0.809	0.869	0.714	0.903	0.616	0.662	0.581	0.097	0.584
	LR	0.826	0.869	0.714	0.903	0.616	0.662	0.581	0.146	0.584
	GBM	0.910	0.886	0.873	0.889	0.632	0.733	0.664	0.146	0.677
	KNN	0.798	0.707	0.698	0.709	0.344	0.461	0.291	0.113	0.325
	NNet	0.821	0.747	0.703	0.751	0.390	0.508	0.359	0.112	0.391
	XGBoost	0.944	0.935	0.746	0.976	0.870	0.803	0.765	0.086	0.768

#### 3.7.2 External validation: performance in an independent temporal cohort

**Figure 7:**
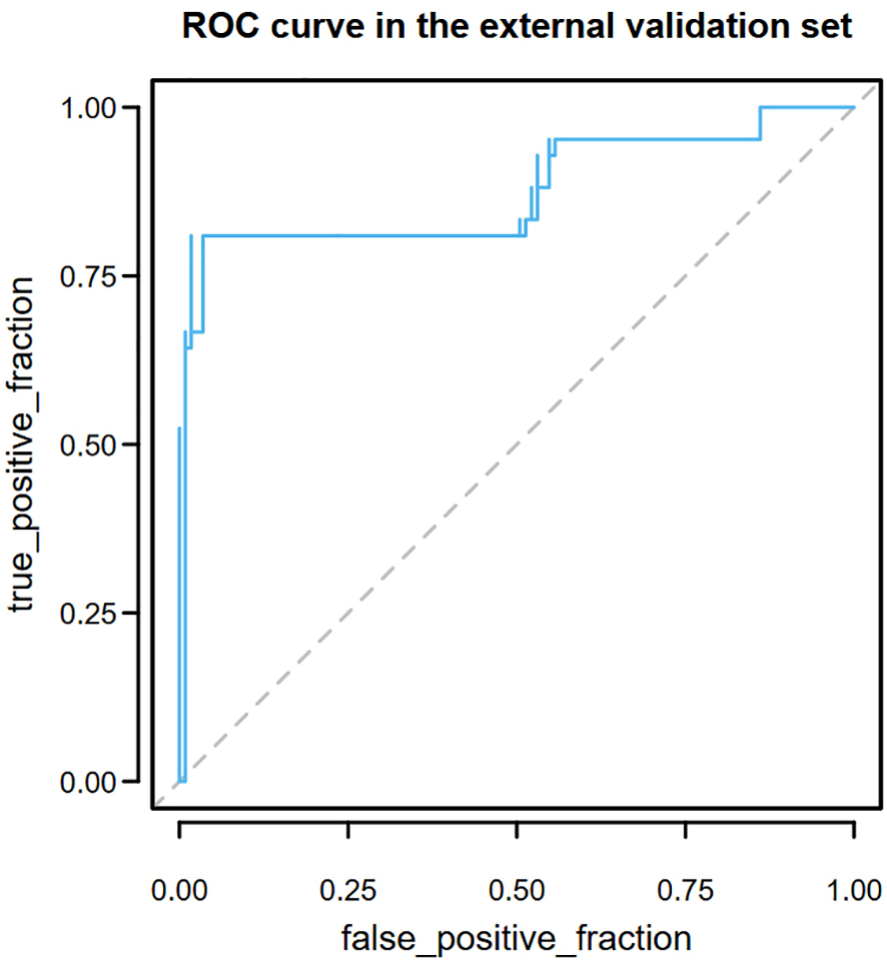
ROC curve of the XGBoost model in the external validation cohort.The ROC curve shows the predictive performance of the XGBoost model in the independent temporal validation cohort. The dashed diagonal line represents the reference line for random prediction.

The XGBoost model was further evaluated in an independent temporal validation cohort from January 2025 to January 2026 to assess its generalizability and clinical utility. The model achieved an AUC of 0.908 (95% CI: 0.845-0.970), with an accuracy of 0.911, sensitivity of 0.833, and specificity of 0.939. **Figure 7** shows the ROC curve for the external validation cohort. **Supplementary Figure 1** shows the DCA for this cohort, indicating clinical net benefit across a range of threshold probabilities. These results show that the model retained good predictive performance in an independent clinical dataset. Because of its stable performance in internal and temporal external validation, the XGBoost model was selected as the final model for development of the online risk calculator.

### 3.8 Stratified ROC analysis of model performance in advanced adenoma and colorectal cancer subgroups

**Figure 8:**
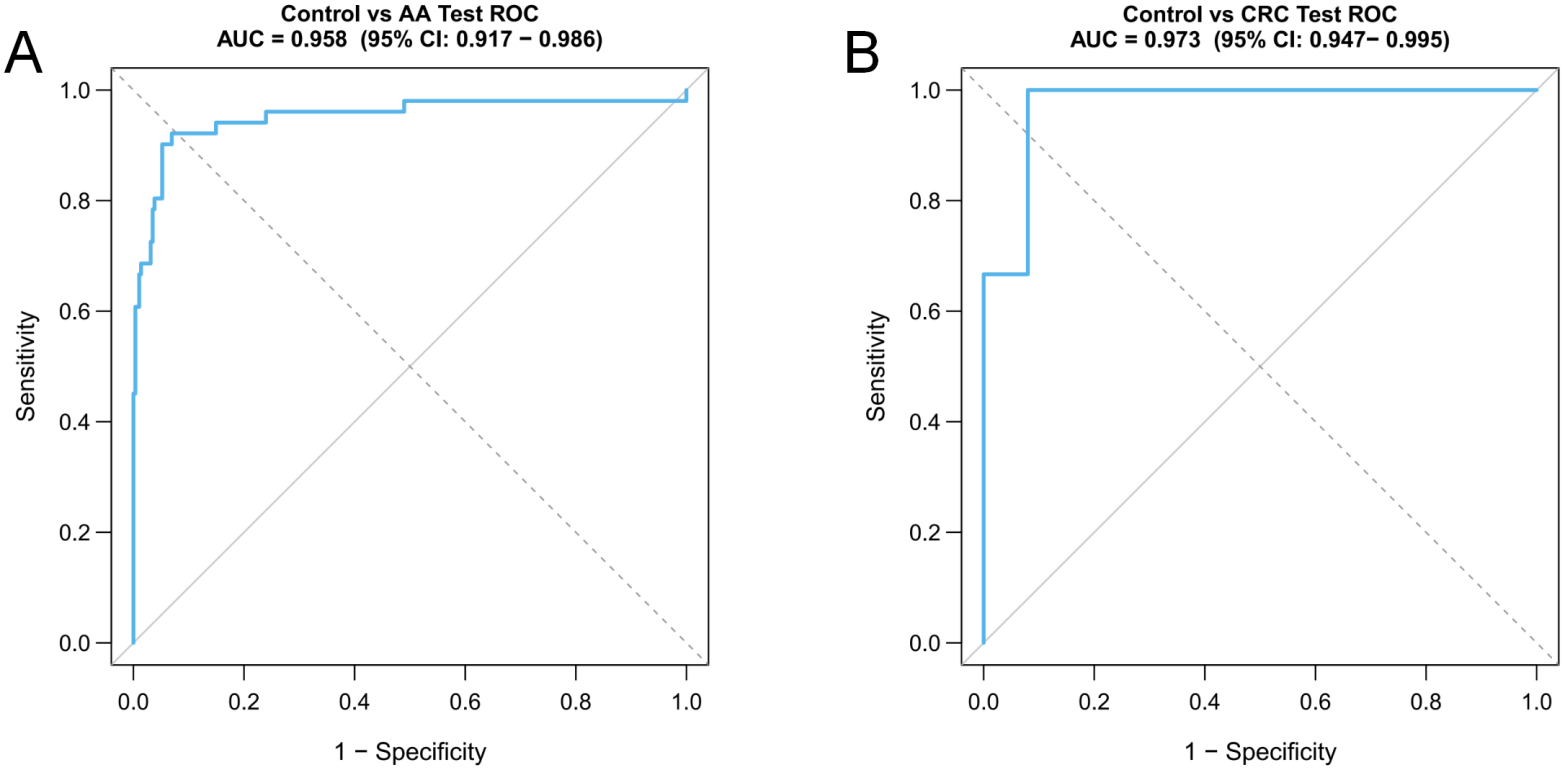
Stratified ROC analysis of the model for distinguishing advanced adenoma and colorectal cancer from controls. **(A)** ROC curve for distinguishing advanced adenoma (AA) from controls. **(B)** ROC curve for distinguishing colorectal cancer (CRC) from controls.

To further investigate whether the high performance of the ACRN model was driven primarily by colorectal cancer (CRC) cases, we performed stratified ROC analyses in AA and CRC subgroups separately. As shown in **Figure 8**, the model demonstrated excellent discriminative performance in both cohorts. In the AA subgroup, the model achieved an AUC of 0.958 (95% CI: 0.917–0.986) for distinguishing AA from controls. In the CRC subgroup, the model yielded an AUC of 0.973 (95% CI: 0.947–0.995) for distinguishing CRC from controls. The consistently high AUC values across these two distinct clinical entities indicate that the model’s superior predictive performance is not attributable solely to the identification of invasive carcinoma, but is equally robust for detecting precancerous advanced adenomas. These findings reinforce the clinical utility of our biomarker panel for early risk stratification across the entire spectrum of advanced colorectal neoplasia and provide strong evidence against the possibility of overestimation driven by cancer cases alone.

### 3.9 Model interpretation using SHAP analysis

**Figure 9:**
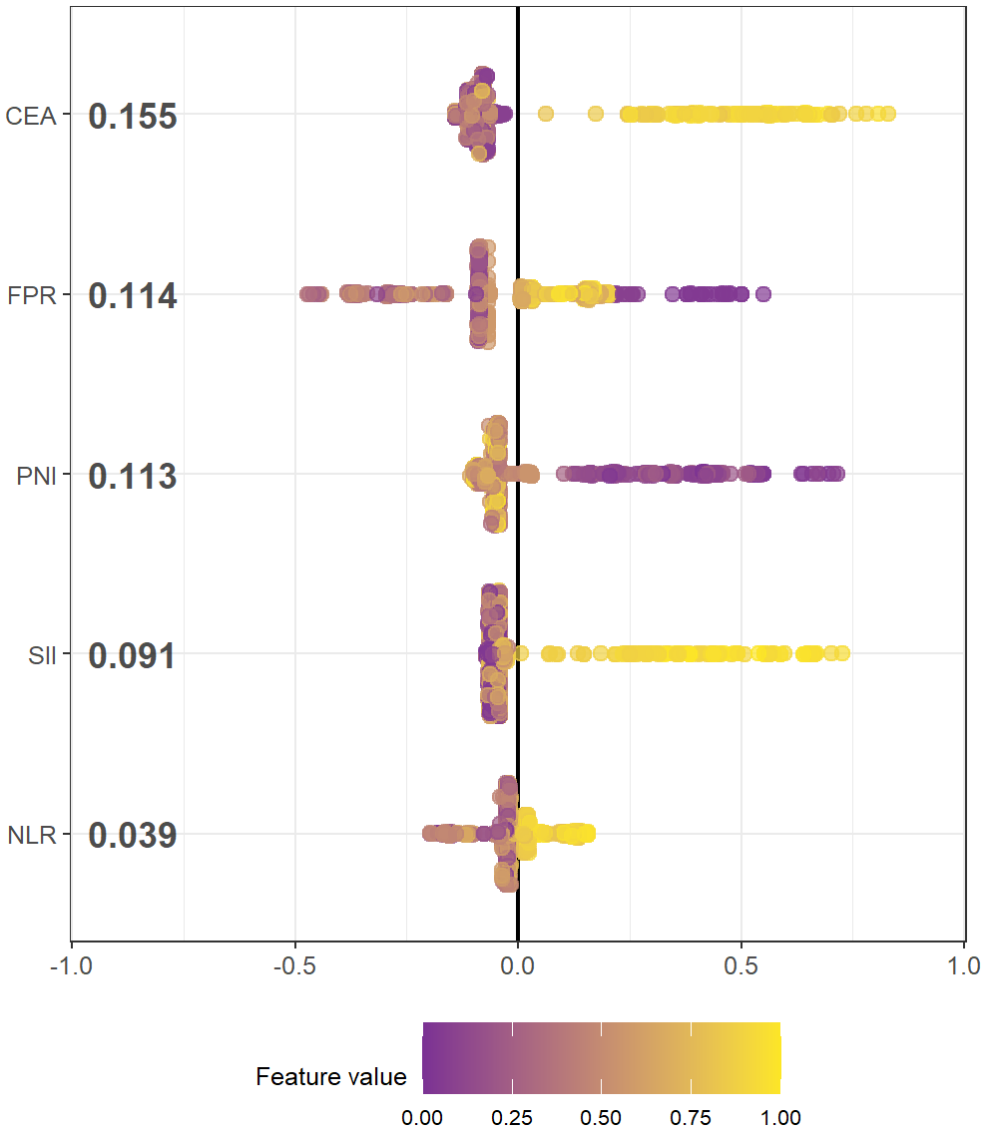
SHAP summary plot of feature importance in the XGBoost model.The SHAP summary plot shows the relative importance of each feature in the XGBoost model. Each dot represents an individual sample, and the color indicates the feature value (yellow: high, purple: low). Features are ranked according to their mean absolute SHAP values. CEA shows the highest contribution to the model prediction, followed by FPR, PNI, SII, and NLR, reflecting their relative importance in predicting advanced colorectal neoplasia.

**Figure 10:**
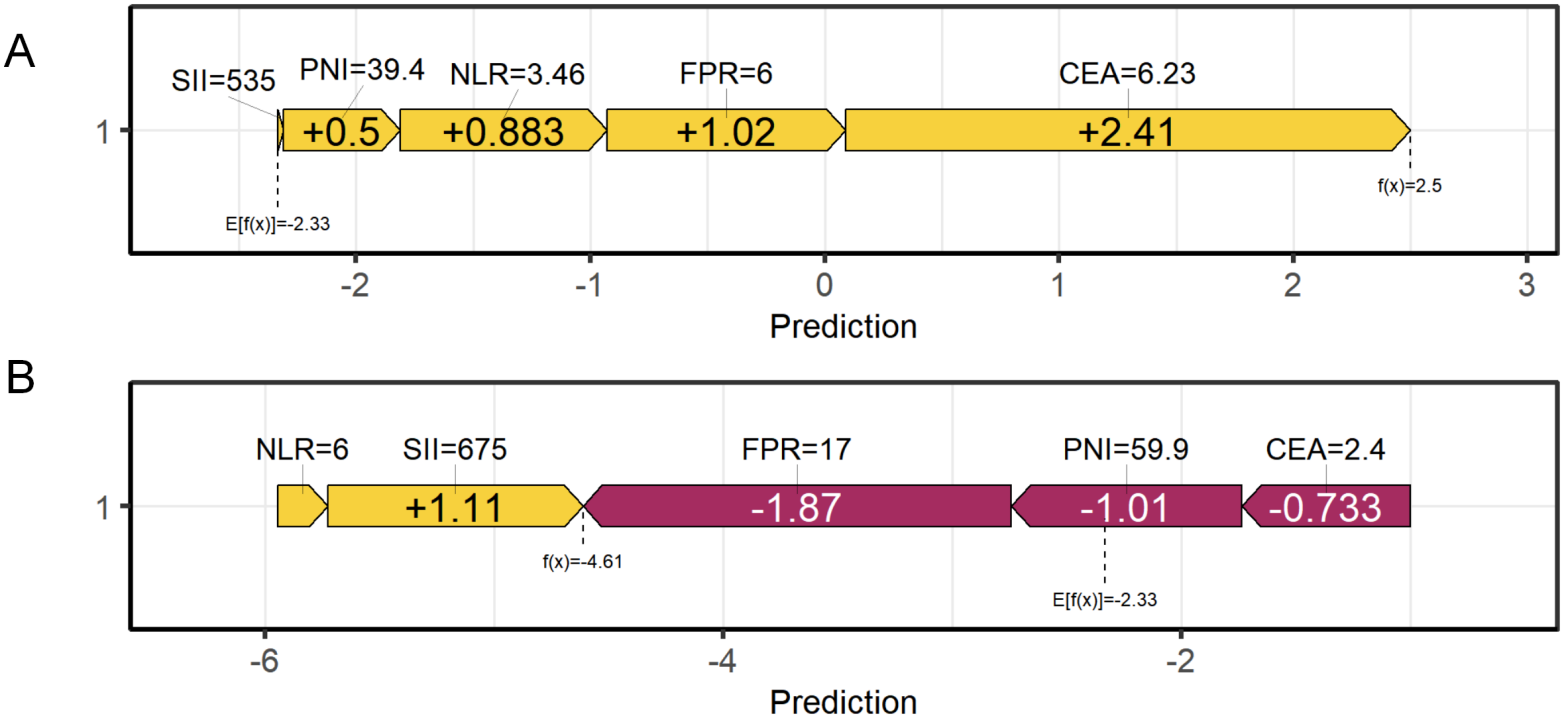
SHAP explanation of the XGBoost model. **(A)** SHAP force plot for a representative high-risk case. **(B)** SHAP force plot for a representative low-risk case. The SHAP force plots illustrate how individual features contribute to the prediction of advanced colorectal neoplasia. Yellow arrows indicate features that increase the predicted risk, whereas purple arrows indicate features that decrease the predicted risk. The contribution of each feature to the final prediction is quantified by the SHAP value.

SHAP analysis was performed to quantify the impact of each feature on the output of the optimal XGBoost model and to improve interpretability. According to the SHAP summary plot (**Figure 9**), CEA was the most important predictor of ACRN risk, followed by FPR, PNI, SII, and NLR. Positive SHAP values were associated with increased CEA and FPR, indicating that higher levels of these biomarkers shifted the model output toward higher ACRN risk. In contrast, higher PNI values contributed negatively to model output, suggesting a potential protective association. SII and NLR had smaller but still meaningful contributions to overall predictive performance. To illustrate individualized prediction, we generated SHAP force plots for representative cases. As shown in **Figure 10**, a typical low-risk case was characterized by relatively low CEA and high PNI, whereas a representative high-risk case was driven by elevated CEA and FPR together with decreased PNI. These examples show how the XGBoost model integrates individual feature contributions to generate personalized risk estimates. Overall, SHAP analysis indicates that tumor burden (CEA) and immuno-inflammatory-nutritional biomarkers (FPR, PNI, SII, and NLR) jointly contribute to XGBoost prediction, with CEA emerging as the dominant feature.

### 3.10 Development of an online risk calculator

**Figure 11:**
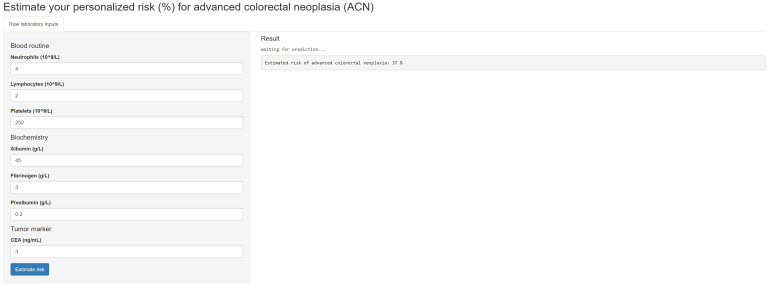
Web-based calculator for individualized risk prediction based on the XGBoost model.

Based on the final XGBoost model, we developed an online risk calculator (https://changchangzhang2001.shinyapps.io/mmonline/) to facilitate clinical implementation (**Figure 11**). In the original study cohort, patients with active infections, chronic inflammatory/autoimmune diseases, or those on immunosuppressants were pre-excluded to minimize confounding effects on peripheral blood biomarkers. The web-based tool has been streamlined to require only routinely available blood tests—neutrophil, lymphocyte, platelet, albumin, and prealbumin levels—from which it automatically computes NLR, SII, PNI, and FPR and generates individualized ACRN risk probabilities in real time. This intuitive platform provides rapid decision support, enabling early identification and risk stratification of advanced colorectal neoplasia while translating a complex machine learning model into a clinically accessible tool that leverages standard laboratory data, thereby enhancing its applicability across diverse clinical settings.

## 4 Discussion

This study systematically evaluated the diagnostic value of systemic inflammatory and nutritional biomarkers for identifying ACRN, including AA and CRC. We developed and validated a machine learning-based prediction model that combined NLR, SII, PNI, FPR, and CEA. Across the training, testing, and independent temporal validation cohorts, XGBoost showed the best overall performance among the algorithms tested. The model also maintained stable predictive ability in the temporal validation dataset. SHAP analysis further clarified the contribution of each variable to model prediction. An online risk calculator was developed to support individualized risk assessment in clinical practice. Together, these findings suggest that inflammatory biomarkers combined with tumor markers may provide a practical and efficient approach for the early detection of ACRN.

There is growing evidence that chronic systemic inflammation significantly contributes to the development and progression of colorectal tumors (18–20). CRC often develops through a series of stages, beginning with normal mucosa, moving to adenoma, and ending in carcinoma (39, 40). During this process, inflammatory responses are closely related to changes in the tumor microenvironment. Persistent inflammation can disrupt intestinal barrier balance and lead to immune dysfunction, creating a microenvironment that favors tumor growth (41, 42). Inflammatory cells and cytokines promote tumor cell proliferation, invasion, and immune escape. Persistent inflammation might elevate oxidative stress and cause DNA damage in epithelial cells, thereby increasing the likelihood of malignant transformation (43, 44). At the same time, systemic inflammation and nutritional status interact with each other during tumor progression. Persistent inflammation increases metabolic demand and reduces nutrient synthesis, which leads to malnutrition and impaired immune function (45). In turn, weakened nutritional and immune status further reduces the body’s ability to remove abnormal cells and precancerous lesions (46–48).

The biomarkers selected in this study reflect different aspects of systemic inflammatory and nutritional status. Prior investigations have found that systemic inflammatory biomarkers, including NLR and SII, are connected to tumor progression and poor prognosis in colorectal cancer, which suggests inflammatory activation and immune suppression (49, 50). FPR reflects the interaction between coagulation and inflammation, while PNI reflects both nutritional status and immune function (51). In this study, NLR, SII, FPR, and CEA gradually increase from the normal group to the AA group and the CRC group, while PNI shows the opposite pattern. These ordered changes suggest that systemic inflammation and nutritional imbalance may occur even at the adenoma stage or before malignant transformation. Such changes can be detected through peripheral blood tests before clear histological cancer develops. This finding has potential clinical value. Traditional screening mainly relies on colonoscopy to detect visible adenomas. In contrast, inflammatory biomarkers may indicate tumor risk at an earlier stage and may help improve screening strategies and follow-up intervals. These results align with earlier research indicating that systemic inflammatory biomarkers vary as colorectal tumors progress.

Compared with previous studies, the present work differs from and extends the existing literature in several important aspects. First, the meta-analysis by Zhang et al. focused on the prognostic role of inflammatory biomarkers in patients with established colorectal cancer (52), whereas our study addresses a fundamentally different clinical question—namely, the diagnostic prediction of advanced colorectal neoplasia (ACRN) in a colonoscopy-based screening cohort, which has direct implications for risk stratification in colorectal cancer screening. Importantly, our findings are consistent with previous meta-analyses demonstrating that systemic inflammatory biomarkers, particularly NLR and SII, are significantly associated with the risk of colorectal neoplasia, further supporting their clinical relevance in colorectal carcinogenesis (53, 54). Second, the diagnostic analysis by Stojkovic Lalosevic et al. was limited to distinguishing colorectal cancer from healthy controls, whereas our study includes both advanced adenoma and colorectal cancer within a unified ACRN composite endpoint, thereby covering the full clinically relevant spectrum from premalignant lesions to invasive carcinoma. Third, the XGBoost model developed in this study demonstrated superior discriminative performance, with an AUC of 0.960 in the training cohort, 0.944 in the testing cohort, and 0.908 in the temporal validation cohort, substantially exceeding the modest discriminatory ability reported in existing risk scoring systems (c-statistics: 0.56–0.65), and also outperforming the combined diagnostic performance reported by Stojkovic Lalosevic et al. for inflammatory biomarkers (AUC = 0.904) (23). Fourth, beyond simple correlation analyses or conventional risk scoring approaches, we established a machine learning-based predictive model incorporating SHAP-based interpretability analysis and further developed a web-based risk calculator, facilitating individualized risk assessment and enhancing its potential for real-world clinical application. In summary, these distinctions—prognostic versus diagnostic modeling, colorectal cancer versus ACRN (including advanced adenoma), limited versus robust discriminative performance, and correlation-based methods versus an interpretable machine learning clinical tool—highlight the innovative contribution of this study within the field of systemic inflammatory biomarkers in colorectal neoplasia.

Over the last few years, there has been a rise in the use of machine learning approaches in cancer prediction and screening models, which have shown promise in detecting high-risk individuals. Global SHAP results show that CEA has the largest contribution to the prediction, followed by FPR and PNI. This finding suggests that inflammatory and nutritional biomarkers provide additional predictive value beyond traditional tumor markers. SHAP force plots at the individual level further show how different biomarkers jointly influence the predicted risk for each patient. These visual explanations help clinicians better understand the model and increase confidence in its predictions.

This study has several strengths. First, we integrate multiple inflammatory and nutritional biomarkers and evaluate their role in colorectal tumor development from the perspective of the inflammation–immune–nutrition axis. Second, both advanced adenoma and colorectal cancer are included in the outcome definition, which covers the full progression spectrum of colorectal tumors. Third, several machine learning algorithms are compared, and a temporal validation cohort is used to assess model stability. This approach improves the methodological reliability of the model. In addition, SHAP analysis enhances model interpretability, and an online calculator is developed to support potential clinical use.

Two additional analytical approaches further strengthen the robustness of our findings. First, we formally evaluated whether common metabolic comorbidities—including diabetes mellitus, cardiovascular disease, and obesity—could confound the observed associations between inflammatory biomarkers and ACRN. In multivariable logistic regression models adjusting for these metabolic factors together with the five selected biomarkers, all five biomarkers remained independently and significantly associated with ACRN, whereas none of the metabolic comorbidities showed a significant association. This indicates that the predictive value of the inflammatory biomarker panel is not substantially confounded by common metabolic conditions, reinforcing its utility as an independent risk stratification tool. Second, to address the concern that the high overall AUC of the combined ACRN model might be predominantly driven by colorectal cancer cases, we performed stratified ROC analyses separately for advanced adenoma and colorectal cancer subgroups. The model achieved comparably high AUC values for distinguishing AA from controls (0.958) and CRC from controls (0.973), demonstrating that the excellent predictive performance is not attributable solely to invasive carcinoma but is equally robust for detecting precancerous advanced adenomas. These stratified results provide strong evidence against overestimation driven by cancer cases alone and support the model’s clinical utility across the entire neoplastic spectrum.

This study has several limitations. First, it was a study conducted at a single center, so selection bias cannot be excluded. Larger multicenter prospective studies are needed to further validate the model and confirm its clinical value. Second, the current model included only routine blood biomarkers. Future studies could incorporate molecular biomarkers or imaging features to further improve predictive accuracy.

## 5 Conclusion

This study demonstrates that an XGBoost model integrating five routine biomarkers—NLR, SII, PNI, FPR, and CEA—achieves excellent performance for detecting advanced colorectal neoplasms (AUC: 0.960, 0.944, and 0.908 in training, testing, and temporal validation cohorts). Stratified analyses confirmed robust discrimination for both advanced adenomas (AUC = 0.958) and colorectal cancer (AUC = 0.973), indicating that the model’s predictive power extends to precancerous lesions rather than being driven solely by invasive carcinoma. The progressive changes of these biomarkers along the normal–adenoma–carcinoma sequence reinforce the biological role of systemic inflammation and nutritional imbalance in colorectal tumorigenesis. The accompanying web-based calculator, requiring only routine blood tests, offers a practical tool for individualized risk stratification and may facilitate early detection in colorectal cancer screening.

## Data Availability

The raw data supporting the conclusions of this article will be made available by the authors, without undue reservation.

## References

[1] Tang S Xie H Kuang J Gao F Gan J Ou H . The value of geriatric nutritional risk index in evaluating postoperative complication risk and long-term prognosis in elderly colorectal cancer patients. Cancer Manag Res. (2020) 12:165–75. doi: 10.2147/CMAR.S234688 32021433 PMC6957008

[2] Wang L Ai M Nie M Zhao L Deng G Hu S . Ehf promotes colorectal carcinoma progression by activating TGF-β1 transcription and canonical TGF-β signaling. Cancer Sci. (2020) 111:2310–24. doi: 10.1111/cas.14444 32372436 PMC7385339

[3] An G Liu Y Hou Y Lei Y Bai J He L . Rrp12 suppresses cell migration and invasion in colorectal cancer cells via regulation of epithelial-mesenchymal transition. J Gastrointest Oncol. (2023) 14:2111–23. doi: 10.21037/jgo-23-254 37969827 PMC10643574

[4] Cenin D Li P Wang J de Jonge L Yan B Tao S . Optimising colorectal cancer screening in Shanghai, China: a modelling study. BMJ Open. (2022) 12:e048156. doi: 10.1136/bmjopen-2020-048156 35577474 PMC9115025

[5] Liu P Liu J Ding M Liu Y Zhang Y Chen X . Fut2 promotes the tumorigenicity and metastasis of colorectal cancer cells via the Wnt/β-catenin pathway. Int J Oncol. (2023) 62. doi: 10.3892/ijo.2023.5483 36734282 PMC9911090

[6] Longobardi S . Colorectal cancer: local results and significance in Hungary. J Gastrointest Oncol. (2024) 15:2552–77. doi: 10.21037/jgo-24-318 39816032 PMC11732334

[7] Sugai T Sugimoto R Eizuka M Osakabe M Yamada S Yanagawa N . Comprehensive analysis of microRNA expression during the progression of colorectal tumors. Dig Dis Sci. (2023) 68:813–23. doi: 10.1007/s10620-022-07576-8 35674995 PMC10011343

[8] Shaukat A Goffredo P Wolf JM Rudser K Church TR . Advanced adenoma and long-term risk of colorectal cancer, cancer-related mortality, and mortality. JAMA Netw Open. (2025) 8:e2459703. doi: 10.1001/jamanetworkopen.2024.59703 39946134 PMC11826353

[9] Dubé C Yakubu M McCurdy BR Lischka A Koné A Walker MJ . Risk of advanced adenoma, colorectal cancer, and colorectal cancer mortality in people with low-risk adenomas at baseline colonoscopy: a systematic review and meta-analysis. Am J Gastroenterol. (2017) 112:1790–801. doi: 10.1038/ajg.2017.360 29087393

[10] He X Hang D Wu K Nayor J Drew DA Giovannucci EL . Long-term risk of colorectal cancer after removal of conventional adenomas and serrated polyps. Gastroenterology. (2020) 158:852–861.e4. doi: 10.1053/j.gastro.2019.06.039 31302144 PMC6954345

[11] Yang Y Han Z Li X Huang A Shi J Gu J . Epidemiology and risk factors of colorectal cancer in China. Chin J Cancer Res. (2020) 32:729–41. doi: 10.21147/j.issn.1000-9604.2020.06.06 33446996 PMC7797231

[12] Liang JQ Wong SH Szeto CH Chu ES Lau HC Chen Y . Fecal microbial DNA markers serve for screening colorectal neoplasm in asymptomatic subjects. J Gastroenterol Hepatol. (2021) 36:1035–43. doi: 10.1111/jgh.15171 32633422 PMC8247299

[13] Yuan Z Chen Y Liu S Yang J Tang C He X . Advances in the study of nucleolar small RNAs in colorectal cancer. iScience. (2026) 29:114789. doi: 10.1016/j.isci.2026.114789 41732269 PMC12924752

[14] Gharebaghi A Afshar S Tapak L Ranjbar H Saidijam M Dinu I . Utilizing an in-silico approach to pinpoint potential biomarkers for enhanced early detection of colorectal cancer. Cancer Inform. (2024) 23:11769351241307163. doi: 10.1177/11769351241307163 39687502 PMC11648020

[15] Calita M Popa P Cherciu Harbiyeli IF Iordache S Ciocalteu A Filip MM . Endocuff-assisted colonoscopy versus standard colonoscopy in colonic polyp detection: experience from a single tertiary centre. Curr Health Sci J. (2021) 47:33–41. doi: 10.12865/chsj.47.01.06 34211745 PMC8200606

[16] Afzal A Aranan YS Roberts T Covington J Vidal L Ahmed S . Diagnostic accuracy of the faecal immunochemical test and volatile organic compound analysis in detecting colorectal polyps: meta-analysis. BJS Open. (2024) 9. doi: 10.1093/bjsopen/zrae154 39972538 PMC11839406

[17] Loktionov A . Biomarkers for detecting colorectal cancer non-invasively: DNA, RNA or proteins? World J Gastrointest Oncol. (2020) 12:124–48. doi: 10.4251/wjgo.v12.i2.124 32104546 PMC7031146

[18] Burgos-Molina AM Téllez Santana T Redondo M Bravo Romero MJ . The crucial role of inflammation and the immune system in colorectal cancer carcinogenesis: a comprehensive perspective. Int J Mol Sci. (2024) 25. doi: 10.3390/ijms25116188 38892375 PMC11172443

[19] Schmitt M Greten FR . The inflammatory pathogenesis of colorectal cancer. Nat Rev Immunol. (2021) 21:653–67. doi: 10.1038/s41577-021-00534-x 33911231

[20] Li Y Luo Y Ran Y Lu F Qin Y . Biomarkers of inflammation and colorectal cancer risk. Front Oncol. (2025) 15:1514009. doi: 10.3389/fonc.2025.1514009 39980561 PMC11839431

[21] Zhu Y Shan D Guo L Chen S Li X . Immune-related lncRNA pairs clinical prognosis model construction for hepatocellular carcinoma. Int J Gen Med. (2022) 15:1919–31. doi: 10.2147/IJGM.S343350 35237066 PMC8882675

[22] Zheng W Wu F Fu K Sun G Sun G Li X . Emerging mechanisms and treatment progress on liver metastasis of colorectal cancer. Onco Targets Ther. (2021) 14:3013–36. doi: 10.2147/OTT.S301371 33986602 PMC8110277

[23] Stojkovic Lalosevic M Pavlovic Markovic A Stankovic S Stojkovic M Dimitrijevic I Radoman Vujacic I . Combined diagnostic efficacy of neutrophil-to-lymphocyte ratio, platelet-to-lymphocyte ratio, and mean platelet volume as biomarkers of systemic inflammation in the diagnosis of colorectal cancer. Dis Markers. (2019) 2019:6036979. doi: 10.1155/2019/6036979 30800188 PMC6360046

[24] Kilincalp S Çoban Ş Akinci H Hamamcı M Karaahmet F Coşkun Y . Neutrophil-to-lymphocyte ratio, platelet-to-lymphocyte ratio, and mean platelet volume as potential biomarkers for early detection and monitoring of colorectal adenocarcinoma. Eur J Cancer Prev. (2015) 24:328–33. doi: 10.1097/CEJ.0000000000000092 25304028

[25] Peng HX Yang L He BS Pan YQ Ying HQ Sun HL . Combination of preoperative NLR, PLR and CEA could increase the diagnostic efficacy for I–III stage colorectal cancer. J Clin Lab Anal. (2017) 31. doi: 10.1002/jcla.22075 27686880 PMC6816914

[26] Olsson M Björkelund AJ Sandberg J Blomberg A Börjesson M Currow D . Factors most strongly associated with breathlessness in a population aged 50–64 years. ERJ Open Res. (2024) 10. doi: 10.1183/23120541.00582-2023 38529345 PMC10962452

[27] Chen D Li G Shu Q Huang J Yuan W Li Y . Superior machine learning model for post-cardiac arrest mortality prediction: a MIMIC-IV cohort study. Resusc Plus. (2026) 28:101223. doi: 10.1016/j.resplu.2026.101223 41717038 PMC12914397

[28] Duarte RB Bernardo WM Sakai CM Silva GL Guedes HG Kuga R . Computed tomography colonography versus colonoscopy for the diagnosis of colorectal cancer: a systematic review and meta-analysis. Ther Clin Risk Manag. (2018) 14:349–60. doi: 10.2147/TCRM.S152147 29503554 PMC5826249

[29] Maida M Camilleri S Manganaro M Garufi S Scarpulla G . New endoscopy advances to refine adenoma detection rate for colorectal cancer screening: none is the winner. World J Gastrointest Oncol. (2017) 9:402–6. doi: 10.4251/wjgo.v9.i10.402 29085566 PMC5648983

[30] Mahmoud R Shah SC Ten Hove JR Torres J Mooiweer E Castaneda D . No association between pseudopolyps and colorectal neoplasia in patients with inflammatory bowel diseases. Gastroenterology. (2019) 156:1333–1344.e3. doi: 10.1053/j.gastro.2018.11.067 30529584 PMC7354096

[31] Qiu H Tang H Yang Y . The non-linear effects of urban green space on promoting physical activity of old adults at different obesity status in semi-arid area: a case study of Lanzhou, China. Geohealth. (2025) 9:e2024GH001273. doi: 10.1029/2024GH001273 40400771 PMC12093153

[32] Wang K Yu G Yan L Lai Y Zhang L . Association of non-traditional lipid indices with diabetes and insulin resistance in US adults: mediating effects of HOMA-IR and evidence from a national cohort. Clin Exp Med. (2025) 25:281. doi: 10.1007/s10238-025-01819-4 40773099 PMC12331762

[33] Yoon S Kwak J Im D Yoon H . Review of outcomes of using lower ethanol concentration (83%) in percutaneous ultrasound-guided renal cyst sclerotherapy in dogs. J Vet Sci. (2023) 24:e61. doi: 10.4142/jvs.23045 37638709 PMC10556289

[34] Semwal R Varadwaj PK . Humdloc: human protein subcellular localization prediction using deep neural network. Curr Genomics. (2020) 21:546–57. doi: 10.2174/1389202921999200528160534 33214771 PMC7604748

[35] Hong D Chang H He X Zhan Y Tong R Wu X . Construction of an early alert system for intradialytic hypotension before initiating hemodialysis based on machine learning. Kidney Dis (Basel). (2023) 9:433–42. doi: 10.1159/000531619 37901708 PMC10601920

[36] Jiang S . Bridging the gap: explainable AI for autism diagnosis and parental support with TabPFNmix and SHAP. Sci Rep. (2025) 15:40850. doi: 10.1038/s41598-025-24662-9 41258065 PMC12630575

[37] Wu W Zhou Z . A comprehensive way to access hospital death prediction model for acute mesenteric ischemia: a combination of traditional statistics and machine learning. Int J Gen Med. (2021) 14:591–602. doi: 10.2147/IJGM.S300492 33658832 PMC7920592

[38] Matsumoto N Yokokawa H Mori H Hiki M Tabe Y Takahashi K . Association between serum zinc concentration levels and severity of coronavirus disease 2019 (COVID-19) in Japanese inpatients. Int J Gen Med. (2024) 17:4745–53. doi: 10.2147/IJGM.S476578 39429954 PMC11491065

[39] Wang M Kong WJ Zhang JZ Lu JJ Hui WJ Liu WD . Association of Helicobacter pylori infection with colorectal polyps and Malignancy in China. World J Gastrointest Oncol. (2020) 12:582–91. doi: 10.4251/wjgo.v12.i5.582 32461789 PMC7235179

[40] van Toledo D JEG IJ Boersma H Musler AR Bleijenberg AGC Dekker E . Polyps and colorectal cancer in serrated polyposis syndrome: contribution of the classical adenoma-carcinoma and serrated neoplasia pathways. Clin Transl Gastroenterol. (2023) 14:e00611. doi: 10.14309/ctg.0000000000000611 37352472 PMC10461936

[41] Zhou J Boutros M . JNK-dependent intestinal barrier failure disrupts host-microbe homeostasis during tumorigenesis. Proc Natl Acad Sci USA. (2020) 117:9401–12. doi: 10.1073/pnas.1913976117 32277031 PMC7196803

[42] Li X Xin S Zheng X Lou L Ye S Li S . Inhibition of the occurrence and development of inflammation-related colorectal cancer by fucoidan extracted from Sargassum fusiforme. J Agric Food Chem. (2022) 70:9463–76. doi: 10.1021/acs.jafc.2c02357 35858119 PMC9354242

[43] Illeperuma RP Kim DK Park YJ Son HK Kim JY Kim J . Areca nut exposure increases secretion of tumor-promoting cytokines in gingival fibroblasts that trigger DNA damage in oral keratinocytes. Int J Cancer. (2015) 137:2545–57. doi: 10.1002/ijc.29636 26076896 PMC4744697

[44] Zhang Y Wang H Wu K Liu Z . Expression of 4-hydroxynonenal in esophageal squamous cell carcinoma. Oncol Lett. (2017) 14:35–40. doi: 10.3892/ol.2017.6127 28693132 PMC5494812

[45] Pan K Jin Y Du W Wang M Zhang Y Liu S . Prognostic value of the neutrophil-to-lymphocyte ratio and C-reactive-protein-to-prealbumin ratio in hospitalized older patients with coronavirus disease 2019. Med (Baltimore). (2024) 103:e37809. doi: 10.1097/MD.0000000000037809 38640293 PMC11029961

[46] Zeng D Wang S Cheng N Liu G Li B . Survival outcomes of preoperative serum biomarkers in patients with surgically treated intrahepatic cholangiocarcinoma. Clin Transl Gastroenterol. (2025) 16:e00845. doi: 10.14309/ctg.0000000000000845 40249087 PMC12180840

[47] Huang J Zhao L Wang K Sun J Tai S Hua R . Controlling nutritional status score evaluates prognosis in patients with non-muscle invasive bladder cancer. Cancer Control. (2021) 28:10732748211021078. doi: 10.1177/10732748211021078 34060373 PMC8204588

[48] Horino T Tokunaga R Miyamoto Y Akiyama T Daitoku N Sakamoto Y . Extracellular water to total body water ratio, a novel predictor of recurrence in patients with colorectal cancer. Ann Gastroenterol Surg. (2024) 8:98–106. doi: 10.1002/ags3.12728 38250685 PMC10797841

[49] Fang J Lin Y Chen Z Lin Y Pan M . The association of inflammatory markers with maternal-neonatal outcome after cervical cerclage. J Inflammation Res. (2023) 16:245–55. doi: 10.2147/JIR.S393666 36698755 PMC9869902

[50] Kang L Liu X Ji W Zheng K Li Y Song Y . Association of neutrophil-to-lymphocyte ratio with nutrition in patients with various types of Malignant tumors: a multicenter cross-sectional study. J Inflammation Res. (2023) 16:1419–29. doi: 10.2147/JIR.S401189 37006808 PMC10064873

[51] Qi Q Song Q Cheng Y Wang N . Prognostic significance of preoperative prognostic nutritional index for overall survival and postoperative complications in esophageal cancer patients. Cancer Manag Res. (2021) 13:8585–97. doi: 10.2147/CMAR.S333190 34815713 PMC8605805

[52] Zhang J Zhang HY Li J Shao XY Zhang CX . The elevated Nlr, Plr and Plt may predict the prognosis of patients with colorectal cancer: A systematic review and meta-analysis. Oncotarget. (2017) 8:68837–46. doi: 10.18632/oncotarget.18575 28978160 PMC5620300

[53] Naszai M Kurjan A Maughan TS . The prognostic utility of pre-treatment neutrophil-to-lymphocyte-ratio (Nlr) in colorectal cancer: A systematic review and meta-analysis. Cancer Med. (2021) 10:5983–97. doi: 10.1002/cam4.4143 34308567 PMC8419761

[54] Gao H Wusiman L Cao BW Wujieke A Zhang WB . The role of preoperative systemic immune-inflammation index in predicting the prognosis of patients with digestive tract cancers: A meta-analysis. Transpl Immunol. (2022) 73:101613. doi: 10.1016/j.trim.2022.101613 35500845

