# Supplementary material for: Systemic inflammatory biomarkers (NLR, SII, PNI and FPR) combined with CEA for predicting advanced colorectal neoplasms: development and temporal validation of a machine learning model
Source: Front Oncol. 2026 Jul 22;16:1832546. doi: 10.3389/fonc.2026.1832546 (PMC13437283; doi:10.3389/fonc.2026.1832546)
Supplement: Supplementary file 1 [file DataSheet1.docx]

Supplementary Material

# 1 Supplementary Figures and Tables

## 1.1 Supplementary Table

**Table S1. Missing data summary of variables**

| Variable | Missing(n) | Missing(%) |
| --- | --- | --- |
| Age | 13 | 0.98% |
| Ca | 0 | 0% |
| Urea | 16 | 1.20% |
| Creatinine | 16 | 1.20% |
| FIB | 0 | 0% |
| AFP | 0 | 0% |
| CEA | 0 | 0% |
| CA199 | 16 | 1.20% |
| CA125 | 28 | 2.11% |
| CA153 | 27 | 2.03% |
| CA724 | 0 | 0% |
| ALT | 0 | 0% |
| AST | 20 | 1.50% |
| TP | 33 | 2.48% |
| ALB | 15 | 1.13% |
| A/G | 45 | 3.38% |
| RBC | 28 | 2.11% |
| Hb | 17 | 1.28% |
| MCHC | 30 | 2.26% |

**Table S2. Baseline characteristics of the temporal validation cohort**

| Variables | non-advanced group(n=115) | Advanced group (42) | P-value |
| --- | --- | --- | --- |
| CEA[Median(Q1-Q3),ng/mL] | 10.00 [1.96, 10.00] | 19.00 [9.00, 33.25] | <0.001 |
| NLR[Median(Q1-Q3)] | 2.00 [1.53, 2.19] | 6.61 [3.76, 7.66] | <0.001 |
| PNI[Median(Q1-Q3)] | 32.00 [30.55, 33.12] | 33.00 [31.96, 35.00] | 0.071 |
| SII[Median(Q1-Q3)] | 334.70 [233.92, 444.00] | 1108.78 [380.46, 1327.50] | <0.001 |
| FPR[Median(Q1-Q3)] | 11.00 [6.00, 14.00] | 25.00 [19.25, 32.75] | <0.001 |

**Table S3. Distribution of metabolic comorbidities between non-advanced and ACRN groups**

| Variable | Level | Outcome = 0 (n = 675) | Outcome = 1 (n = 147) | P value |
| --- | --- | --- | --- | --- |
| DM[n,(%)] | No | 536 (79.4%) | 113 (76.9%) | 0.494 |
|  | Yes | 139 (20.6%) | 34 (23.1%) |  |
| CVD[n,(%)] | No | 427 (63.3%) | 94 (63.9%) | 0.876 |
|  | Yes | 248 (36.7%) | 53 (36.1%) |  |
| BMI[n,(%)] | Underweight | 312 (46.2%) | 57 (38.8%) | 0.253 |
|  | Normal | 324 (48.0%) | 81 (55.1%) |  |
|  | Overweight | 39 (5.8%) | 9 (6.1%) |  |

**1.2	Supplementary Figure**

Figure S1.DCA of the XGBoost model in the external validation cohort. The blue line represents the net benefit of the XGBoost model across a range of threshold probabilities. The black line indicates the “treat-all” strategy, and the gray line represents the “treat-none” strategy. The results demonstrate that the XGBoost model provides a higher net clinical benefit over a wide range of threshold probabilities compared with the default strategies.
